# Complete genome sequence of *Eggerthella lenta* type strain (IPP VPI 0255^T^)

**DOI:** 10.4056/sigs.33592

**Published:** 2009-09-28

**Authors:** Elizabeth Saunders, Rüdiger Pukall, Birte Abt, Alla Lapidus, Tijana Glavina Del Rio, Alex Copeland, Hope Tice, Jan-Fang Cheng, Susan Lucas, Feng Chen, Matt Nolan, David Bruce, Lynne Goodwin, Sam Pitluck, Natalia Ivanova, Konstantinos Mavromatis, Galina Ovchinnikova, Amrita Pati, Amy Chen, Krishna Palaniappan, Miriam Land, Loren Hauser, Yun-Juan Chang, Cynthia D. Jeffries, Patrick Chain, Linda Meincke, David Sims, Thomas Brettin, John C. Detter, Markus Göker, Jim Bristow, Jonathan A. Eisen, Victor Markowitz, Philip Hugenholtz, Nikos C. Kyrpides, Hans-Peter Klenk, Cliff Han

**Affiliations:** 1 DOE Joint Genome Institute, Walnut Creek, California, USA; 2 DSMZ - German Collection of Microorganisms and Cell Cultures GmbH, Braunschweig, Germany; 3 Los Alamos National Laboratory, Bioscience Division, Los Alamos, New Mexico, USA; 4 Biological Data Management and Technology Center, Lawrence Berkeley National Laboratory, Berkeley, California, USA; 5 Oak Ridge National Laboratory, Oak Ridge, Tennessee, USA; 6 Lawrence Livermore National Laboratory, Livermore, California, USA; 7 University of California Davis Genome Center, Davis, California, USA

**Keywords:** mesophile, anaerobic, human intestinal microflora, pathogenic, bacteremia, Gram-positive, *Coriobacteriaceae*

## Abstract

*Eggerthella lenta* (Eggerth 1935) Wade *et al.* 1999, emended Würdemann *et al*. 2009 is the type species of the genus *Eggerthella*, which belongs to the actinobacterial family *Coriobacteriaceae*. *E. lenta* is a Gram-positive, non-motile, non-sporulating pathogenic bacterium that can cause severe bacteremia. The strain described in this study has been isolated from a rectal tumor in 1935. Here we describe the features of this organism, together with the complete genome sequence, and annotation. This is the first complete genome sequence of the genus *Eggerthella*, and the 3,632,260 bp long single replicon genome with its 3123 protein-coding and 58 RNA genes is part of the *** G****enomic* *** E****ncyclopedia of* *** B****acteria and* *** A****rchaea * project.

## Introduction

Strain VPI 0255^T^ (= DSM 2243 = ATCC 25559 = JCM 9979) is the type strain of the species *Eggerthella lenta*, which was first described in 1935 by Eggerth as ‘*Bacteroides lentus*’ [1], later in 1938 renamed by Prévot in ‘*Eubacterium lentum*’ [2], and was also known under the synonym ‘*Pseudobacterium lentum*’ Krasil’nikov 1949 [3]. The strain has been described in detail by Moore *et al*. in 1971 [4]. Based on 16S rRNA sequence divergence and the presence of unique phenotypic characters the strain was then transferred to the new genus *Eggerthella* as *E. lenta* (Kageyama *et al.* 1999, Wade *et al*. 1999 [5,6] In 2004 two novel *Eggerthella* species, *E. hongkongensis* and *E. sinensis* were characterized and described in addition [7]. Recently, *E. hongkongensis* was reclassified as *Paraeggerthella hongkongensis* [8]. Although the two *Eggerthella* species and *P. hongkongensis* are part of the human gut flora, they can be the agent of severe bacteremia. So far the pathogenic potential of the genera are poorly analyzed [7]. Here we present a summary classification and a set of features for *E. lenta* VPI 0255^T^, together with the description of the complete genomic sequencing and annotation.

## Classification and features

Members of the species *E. lenta* have been isolated from several abscesses, from appendix tissues, peritoneal fluid and intestinal tumors. The organism is often involved in mixed infections with less fastidious bacteria. Difficulties in cultivation and identification are probably the reason why bacteremia caused by *Eggerthella* is rarely reported. Half of the cases of *Eggerthella* bacteremia are induced by the two novel species: *E. sinensis* and *P*. *hongkongensis* [7]. Stinear *et al*. described an isolate (AF304434) from human feces resembling *E. lenta* (98% identity) that carries an enterococcal *vanB* resistance locus probably received via lateral gene transfer or as a result of genetic mutations [9]. Clavel *et al*. investigated the occurrence and activity of dietary lignans activating bacterial communities in human feces and identified an *E. lenta* strain (AY937380) with 98.2% sequence similarity to strain VPI 0255^T^ [10]. Lignans are a class of phytoestrogen which can be metabolized to the biologically active enterolignans, enterodiol and enterolactone. The human intestinal microbiota is essential for the conversation of the dietary lignans *e.g.* secoisolariciresinol diglucoside *via* secoisolariciresinol (SECO) to the enterolignans. Clavel and co-workers also reported that the dehydroxylation of SECO is catalyzed by *Eggerthella lenta* [11]. Based on 16S rRNA gene sequence analyses another five uncultured clones with 99% identity to *E. lenta* were reported at the NCBI BLAST server (status June 2009). These clones were derived from the analyses of feces samples from humans. *e.g*. associated with obesity [12,13], but also from marine metagenomes [14]

**Figure 1:**
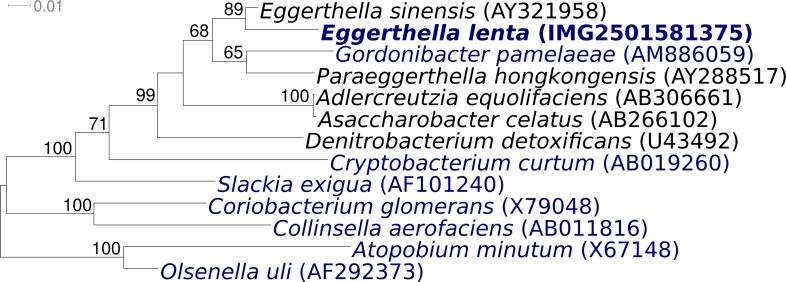
Phylogenetic tree of *E. lenta* strain VPI 0255^T^ and all type strains of the genus *Eggerthella* as well as the type strains from all other genera of the family *Coriobacteriaceae* inferred from 1,373 aligned characters [15,16] of the 16S rRNA gene under the maximum likelihood criterion [17]. The branches are scaled in terms of the expected number of substitutions per site. Numbers above branches are support values from 1,000 bootstrap replicates if larger than 60%. Lineages with type strain genome sequencing projects registered in GOLD [18] are shown in blue, published genomes in bold, including two of which are reported in this issue of *SIGS* [19,20]

Figure 1 shows the phylogenetic neighborhood of *E. lenta* strain VPI 0255^T^ in a 16S rRNA based tree. The sequences of the three identical copies of the 16S rRNA gene in the genome differ by three nucleotides from the previously published 16S rRNA sequence generated from ATCC 25559 (AF292375). The slight difference between the genome data and the reported 16S rRNA gene sequence is most likely due to sequencing errors in the previously reported sequence data.

**Figure 2:**
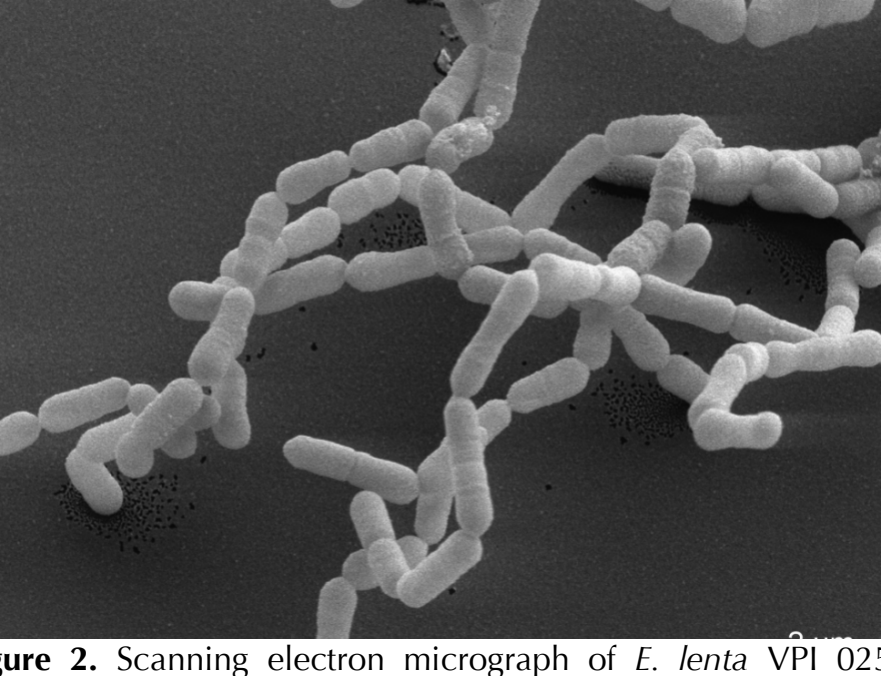
Scanning electron micrograph of *E. lenta* VPI 0255^T^ (Manfred Rohde, Helmholtz Centre for Infection Biology, Braunschweig)

*E. lenta* strain VPI 0255^T^ was originally isolated from a rectal tumor and described as Gram-positive, non-motile and non-sporulating (Table 1) [1]. Cells are rod shaped and occur singly or in long chains up to 20 elements (Figure 2). The cell size and morphology varies depending on the substrate and the age of the culture. Surface colonies were described as circular to slightly scalloped, convex, shiny, gray and translucent. *E. lenta* is obligately anaerobic and its optimal growth temperature is 37° C [4]. Growth is stimulated by arginine. The existence of the arginine dihydrolase pathway as an important energy source was described by Sperry and Wilkens in 1976 [26]. *E. lenta* is asaccharolytic [4,26,29], Gelatin is not liquefied, aesculin is not hydrolyzed and nitrate is reduced [29]. *E. lenta* is bile-resistant and primarily found in human feces [6].

**Table 1:** Classification and general features of *B. cavernae* HKI 0122^T^ in accordance with the MIGS recommendations [21]

**MIGS ID**	**Property**	**Term**	**Evidence code**
	Classification	Domain *Bacteria*	TAS [22]
		Phylum *Actinobacteria*	TAS [23]
		Class *Actinobacteria*	TAS [24]
		Order *Coriobacteriales*	TAS [24]
		Suborder “*Coriobacterineae”*	TAS [25]
		Family *Coriobacteriaceae*	TAS [24]
		Genus *Eggerthella*	TAS [6]
		Species *Eggerthella lenta*	TAS [6]
		Type strain VPI 0255	
	Gram stain	positive	TAS [1,4]
	Cell shape	rods, single or arranged in pairs and chains	TAS [1,4]
	Motility	non-motile	TAS [1,4]
	Sporulation	non-sporulating	TAS [1,4]
	Temperature range	mesophile	TAS [4]
	Optimum temperature	37°C	TAS [4]
	Salinity	6.5% NaCl, poor to moderate growth	TAS [4]
MIGS-22	Oxygen requirement	anaerobic	TAS [1,4]
	Carbon source	arginine	TAS [24,26]
	Energy source	arginine	TAS [26]
MIGS-6	Habitat	blood, human intestinal microflora	TAS [1,7]
MIGS-15	Biotic relationship	free living	NAS
MIGS-14	Pathogenicity	bacteremia	TAS [27]
	Biosafety level	2	TAS [28]
	Isolation	rectal tumor	TAS [1,29]
MIGS-4	Geographic location	not reported	
MIGS-5	Sample collection time	1938	TAS [1]
MIGS-4.1 MIGS-4.2	Latitude – Longitude	not reported	
MIGS-4.3	Depth	not reported	
MIGS-4.4	Altitude	not reported	

Evidence codes - IDA: Inferred from Direct Assay (first time in publication); TAS: Traceable Author Statement (i.e., a direct report exists in the literature); NAS: Non-traceable Author Statement (i.e., not directly observed for the living, isolated sample, but based on a generally accepted property for the species, or anecdotal evidence). These evidence codes are from the Gene Ontology project [30]. If the evidence code is IDA, then the property was directly observed for a living isolate by one of the authors, or an expert or reputable institution mentioned in the acknowledgements.

### Chemotaxonomy

The cell wall of *E. lenta* strain VPI 0255^T^ contains A1γ-type peptidoglycan glutamic acid occurred in D-form and diaminopimelic acid in *meso* configuration. Mycolic acids and teichonic acids were not reported. Strain VPI 0255^T^ contains menaquinone MK-6 as the major respiratory lipoquinone (63.7%) and a lower amount of the methylmenaquinone MMK-6 (36.3%) [8,29,31]. As the predominant fatty acids the unbranched saturated 16:0 DMA (29.4%) and the monounsaturated fatty acid 18:1w9c (22.0%) were identified [5,6]. Polar lipids consist of two phospholipids, phosphatidylglycerol and diphosphatidylglycerol, and four glycolipids GL1-GL4 [8].

## Genome sequencing and annotation

### Genome project history

This organism was selected for sequencing on the basis of each phylogenetic position, and is part of the *** G****enomic* *** E****ncyclopedia of* *** B****acteria and* *** A****rchaea * project. The genome project is deposited in the Genome OnLine Database [18] and the complete genome sequence in GenBank. Sequencing, finishing and annotation were performed by the DOE Joint Genome Institute (JGI). A summary of the project information is shown in Table 2.

**Table 2:** Genome sequencing project information

**MIGS ID**	**Property**	**Term**
MIGS-31	Finishing quality	Finished
MIGS-28	Libraries used	Three genomic libraries: two Sanger libraries - 8 kb pMCL200 and fosmid pcc1Fos – and one 454 pyrosequence standard library
MIGS-29	Sequencing platforms	ABI3730, 454 GS FLX
MIGS-31.2	Sequencing coverage	10.2× Sanger; 25.3× pyrosequence
MIGS-30	Assemblers	Newbler version 1.1.02.15, phrap
MIGS-32	Gene calling method	Prodigal, GenePRIMP
	Genbank ID	CP001726
	Genbank Date of Release	September 9, 2009
	GOLD ID	Gc01054
	NCBI project ID	21093
	Database: IMG-GEBA	2501533210
MIGS-13	Source material identifier	DSM 2243
	Project relevance	Tree of Life, GEBA

### Growth conditions and DNA isolation

*E. lenta* strain VPI 0255^T^, DSM 2243, was grown anaerobically in DSMZ medium 209 (*Eubacterium lentum* Medium [32]) at 37°C. DNA was isolated from 1-1.5 g of cell paste using Qiagen Genomic 500 DNA Kit (Qiagen, Hilden, Germany) following the manufacturer’s protocol without modifications.

### Genome sequencing and assembly

The genome was sequenced using a combination of Sanger and 454 sequencing platforms. All general aspects of library construction and sequencing can be found at the JGI website. 454 Pyrosequencing reads were assembled using the Newbler assembler version 1.1.02.15 (Roche). Large Newbler contigs were broken into 4,901 overlapping fragments of 1,000 bp and entered into the assembly as pseudo-reads. The sequences were assigned quality scores based on Newbler consensus q-scores with modifications to account for overlap redundancy and to adjust inflated q-scores. A hybrid 454/Sanger assembly was made using the parallel phrap assembler (High Performance Software, LLC). Possible mis-assemblies were corrected with Dupfinisher or transposon bombing of bridging clones [33]. Gaps between contigs were closed by editing in Consed, custom primer walk or PCR amplification. A total of 358 Sanger finishing reads were produced to close gaps, to resolve repetitive regions, and to raise the quality of the finished sequence. The final assembly consists of 39,464 Sanger and 471,609 pyrosequence (454) reads. Together all sequence types provided 35.5x coverage of the genome. The error rate of the completed genome sequence is less than 1 in 100,000.

### Genome annotation

Genes were identified using Prodigal [34] as part of the Oak Ridge National Laboratory genome annotation pipeline, followed by a round of manual curation using the JGI GenePRIMP pipeline [35]. The predicted CDSs were translated and used to search the National Center for Biotechnology Information (NCBI) nonredundant database, UniProt, TIGRFam, Pfam, PRIAM, KEGG, COG, and InterPro databases. Additional gene prediction analysis and functional annotation was performed within the Integrated Microbial Genomes Expert Review (IMG-ER) platform [36].

## Genome properties

**Figure 3:**
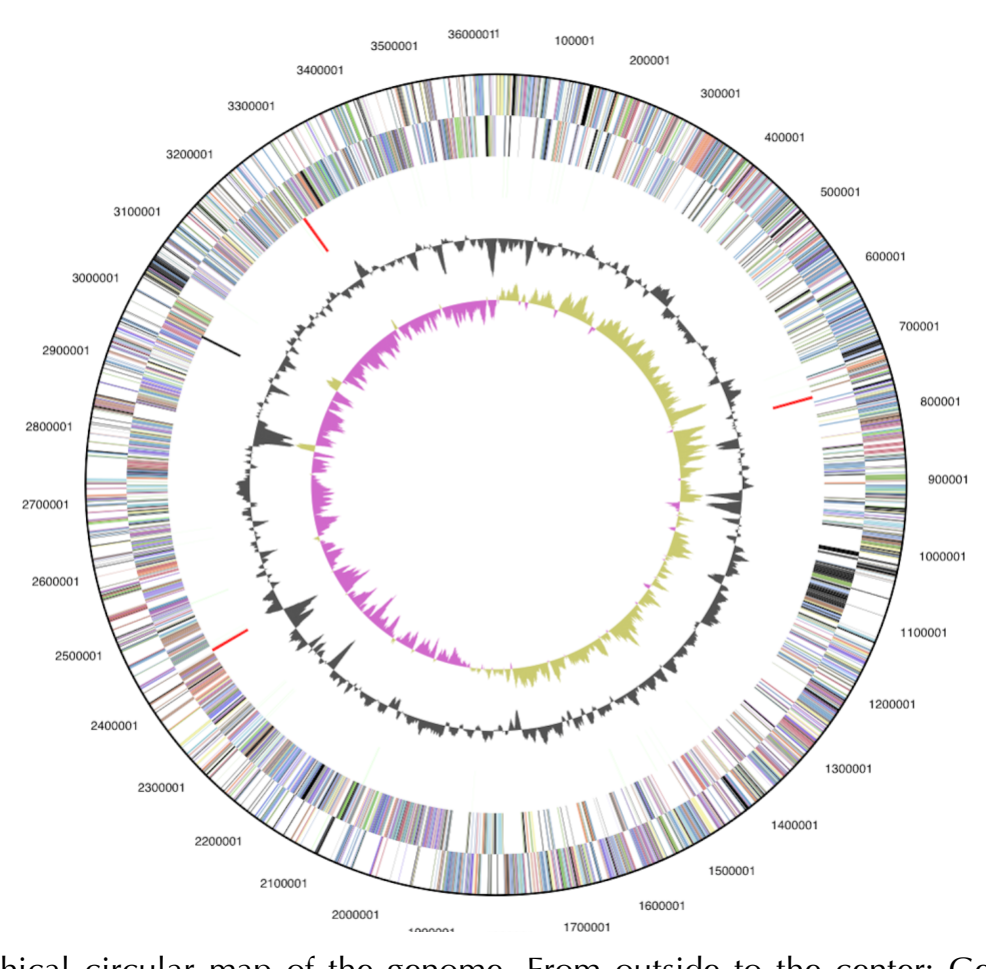
Graphical circular map of the genome. From outside to the center: Genes on forward strand (color by COG categories), Genes on reverse strand (color by COG categories), RNA genes (tRNAs green, rRNAs red, other RNAs black), GC content, GC skew.

The genome is 3,632,260 bp long and comprises one main circular chromosome with a 64.2% GC content (Table 3 and Figure 3). Of the 3,181 genes predicted, 3,123 were protein coding genes, and 58 RNAs. 53 pseudogenes were also identified. A majority of the genes (70.9%) were assigned with a putative function while the remaining genes were annotated as hypothetical proteins. The properties and the statistics of the genome are summarized in Table 3. The distribution of genes into COGs functional categories is presented in Table 4.

**Table 3:** Genome Statistics

**Attribute**	**Value**	**% of Total**
Genome size (bp)	3,632,260	100.00%
DNA Coding region (bp)	3,211,405	88.41%
DNA G+C content (bp)	2,322,078	64.20%
Number of replicons	1	
Extrachromosomal elements	0	
Total genes	3,181	100.00%
RNA genes	58	1.67%
rRNA operons	3	
Protein-coding genes	3,123	98.18%
Pseudo genes	53	1.67%
Genes with function prediction	2,255	70.89%
Genes in paralog clusters	629	19.77%
Genes assigned to COGs	2285	71.83%
Genes assigned Pfam domains	2316	72.81%
Genes with signal peptides	781	24.55%
Genes with transmembrane helices	990	31.12%
CRISPR repeats	1	

**Table 4:** Number of genes associated with the general COG functional categories

**Code**	**Value**	**%age**	**Description**
J	142	4.5	Translation, ribosomal structure and biogenesis
A	0	0.0	RNA processing and modification
K	310	9.9	Transcription
L	130	4.2	Replication, recombination and repair
B	0	0.0	Chromatin structure and dynamics
D	25	0.8	Cell cycle control, mitosis and meiosis
Y	0	0.0	Nuclear structure
V	80	2.6	Defense mechanisms
T	201	6.4	Signal transduction mechanisms
M	129	4.1	Cell wall/membrane biogenesis
N	13	0.4	Cell motility
Z	0	0.0	Cytoskeleton
W	0	0.0	Extracellular structures
U	51	1.6	Intracellular trafficking and secretion
O	81	2.6	Posttranslational modification, protein turnover, chaperones
C	293	9.4	Energy production and conversion
G	79	2.5	Carbohydrate transport and metabolism
E	180	5.8	Amino acid transport and metabolism
F	60	1.9	Nucleotide transport and metabolism
H	89	2.8	Coenzyme transport and metabolism
I	69	2.2	Lipid transport and metabolism
P	132	4.2	Inorganic ion transport and metabolism
Q	32	1.0	Secondary metabolites biosynthesis, transport and catabolism
R	262	8.4	General function prediction only
S	195	6.2	Function unknown
-	838	26.8	Not in COGs
